# Pilot of asymptomatic swabbing of humans following exposures to confirmed avian influenza A(H5) in avian species in England, 2021/2022

**DOI:** 10.1111/irv.13187

**Published:** 2023-08-23

**Authors:** Fernando Capelastegui, Julianna Smith, Jharna Kumbang, Clare Humphreys, Simon Padfield, Jonathan Turner, Andrew Mumford, Nick Richardson, Isabel Oliver, Gavin Dabrera

**Affiliations:** ^1^ UK Health Security Agency London UK

**Keywords:** A(H5N1), asymptomatic testing, avian influenza, emerging disease, England, surveillance

## Abstract

A programme of asymptomatic swabbing was piloted in 2021/2022 in England to further understand the risk of human infection with avian influenza in exposed individuals and to evaluate this surveillance approach as a public health measure. There were challenges in deploying this pilot that will need to be addressed for future seasons. However, there was one detection of avian influenza A(H5N1) in a human despite low uptake in eligible exposed persons. Future use of asymptomatic swabbing could help provide an evidence base to quantify asymptomatic infection, quickly identify signals of increased animal to human transmission and improve public health preparedness.

## 1 INTRODUCTION

There have been unprecedented and increasing levels of avian influenza (AI) detected in bird species across England and globally since 2020,^1^ particularly A(H5N1) of clade 2.3.4.4b.^2^, ^3^ AI viruses such as A(H5N1) have been previously associated with severe human disease in infected persons,^4^ though generally, infections have been sporadic and principally focussed among those who are directly exposed to infected birds.^5^

In England, AI detections in birds confirmed by APHA (Animal and Plant Health Agency) are notified to UKHSA (UK Health Security Agency) Health Protection Teams (HPTs) who undertake public health actions^6^, ^7^ including identification of exposed persons (EPs), provision of advice on PPE (personal protective equipment), and for specific haemagglutinin subtypes including H5, H7 or H9, antiviral prophylaxis and health monitoring.^7^ PCR testing is recommended in EPs who develop symptoms. However, the detection of A(H5N8) clade 2.3.4.4b in exposed poultry workers in Russia demonstrated the potential for asymptomatic infection.^8^, ^9^

In the context of an increasing number of bird detections^1^ and consequent risk of exposure, a surveillance pilot study was developed in England to enhance human case finding and understand asymptomatic infection. The primary aim was to determine the feasibility of asymptomatic AI surveillance in EPs to inform future public health guidance. This offer was in addition to the existing recommendations for antiviral prophylaxis, health monitoring and PPE use. The pilot operated during the height of the 2021/2022 season in England and involved collection of a single upper respiratory swab for testing from EPs within 10 days of last unprotected exposure.

This paper describes the characteristics of EPs who participated in the surveillance to inform future development of asymptomatic swabbing.

## 2 METHODS

### 2.1 Data sources and collection

HPTs identified eligible EPs:

within 10 days of last direct exposure to laboratory confirmed incidents in avian species.

AND

not used full PPE or reported a breach in PPE during the exposure.

Once HPTs had agreement and consent from the EP, swab kit delivery and collection was organised. Samples were collected in viral transport media and returned in secure postal packaging for testing at UKHSA laboratories using real‐time PCR assays to detect influenza A and H5.

Nasal‐pharyngeal swabs were collected through self‐swabbing or rarely by a health professional at home or away from the infected site, to avoid the risk of environmental contamination. This offer did not replace testing following the development of influenza compatible symptoms during the follow‐up period.

Surveillance forms were completed for each incident, routinely collated within UKHSA and processed into a secure dataset. Individual person exposure records were linked to UKHSA laboratory respiratory testing information. Person exposures included in this analysis were those who met the eligibility criteria above between 01 December 2021 and 31 March 2022 and had recorded data including incident type, EP role, region, symptom status, use of PPE and antivirals, swabbing status and result for each person exposure (some EPs had multiple separate exposures).

### 2.2 Data analysis

Due to small numbers in the EP occupational category data, roles were aggregated to disease control and roles not in disease control. Roles within disease control (i.e., APHA staff, contractors, farm workers and veterinary staff) are involved with investigation, decontamination and culling with expected frequent exposure to AI. Roles not typically involved with disease control include members of the public and nature reserve workers. The regions of London and the South East were combined due to small numbers. Age and sex are not routinely collected so could not be included.

Descriptive analysis of eligible person exposures (due to the possibility of individuals having multiple exposures) was carried out to summarise exposure characteristics (Table 1).

**TABLE 1:** Demographic and exposure characteristics of eligible exposure events.

	Not swabbed	Swabbed	Total (percentage of total eligible cohort)
Swabbing status	187 (69.00%)	84 (31.00%)	271 (100%)
Type of incident			
Infected premise	80 (60.61%)	52 (39.39%)	132 (48.71%)
Wild bird	107 (76.98%)	32 (23.02%)	139 (51.29%)
Role type			
Disease control	84 (64.12%)	47 (35.88%)	131 (48.34%)
Other	93 (72.66%)	35 (27.34%)	128 (47.23%)
Not known	10 (83.33%)	2 (16.67%)	12 (4.43%)
Region			
East Midlands	38 (71.70%)	15 (28.30%)	53 (19.56%)
East of England	25 (58.14%)	18 (41.86%)	43 (15.87%)
London and South East	19 (90.48%)	2 (9.52%)	21 (7.75%)
North East	6 (66.67%)	3 (33.33%)	9 (3.32%)
North West	65 (73.03%)	24 (26.97%)	89 (32.84%)
South West	6 (33.33%)	12 (66.67%)	18 (6.64%)
West Midlands	10 (52.63%)	9 (47.37%)	19 (7.01%)
Yorkshire and Humber	18 (94.74%)	1 (5.26%)	19 (7.01%)
Received antiviral prophylaxis			
No	126 (87.50%)	18 (12.50%)	144 (53.14%)
Unknown	1 (50.00%)	1 (50.00%)	2 (0.74%)
Yes	60 (48.00%)	65 (52.00%)	125 (46.13%)
Swab result			
Influenza (H5)	‐	1 (1.19%)	‐
Negative Influenza A PCR	‐	64 (76.19%)	‐
Testing offered but result not received	‐	19 (22.62%)	‐

Univariate logistic regression was used to assess associations between asymptomatic swabbing and the following: region, incident type, EP role and receipt of antiviral prophylaxis (Table 2).

**TABLE 2:** Single variable model results of exposure and demographic characteristics versus asymptomatic swabbing uptake.

Characteristic	*N* (where data complete)	OR	95% CI	*p*‐value
Role	259			
Disease control		Reference group		
Other		0.67	0.39, 1.14	0.14
Received prophylaxis	269			
No		Reference group		
Yes		7.58	4.21, 14.2	<0.001
Region	271			
East Midlands		Reference group		
East of England		1.82	0.78, 4.32	0.17
London and South East		0.27	0.04, 1.08	0.1
North East		1.27	0.24, 5.49	0.76
North West		0.94	0.44, 2.03	0.86
South West		5.07	1.66, 17.0	0.006
West Midlands		2.28	0.77, 6.81	0.14
Yorkshire and Humber		0.14	0.01, 0.78	0.067
Incident type	271			
Infected premise		Reference group		
Wild bird		0.46	0.27, 0.78	0.004

Abbreviations: CI, confidence interval; OR, odds ratio.

## 3 RESULTS

Exposure information was available for 219 confirmed A(H5) incidents: 66 infected premises (IP) and 153 wild bird (WB) incidents, which occurred in England between 01 December 2021 and 31 March 2022.

A total of 1617 exposures was reported during this period (includes participants with multiple exposures). Of these, 84 out of 271 eligible participants agreed to enrol (31%). The number of exposures eligible for asymptomatic swabbing was similar between IP and WB detections (132 and 139, respectively). Of those swabbed, 52 were associated with IPs (39.4% of eligible exposures), and 32 were associated with WB detections (23.0% of eligible exposures).

Asymptomatic swabbing uptake in occupations with primary roles in disease control was 35.9% with 47 swabs taken; 35 swabs were taken in the rest, representing 27.3% of exposures in this group.

Uptake varied regionally with the highest proportion being in the South West of England with 67% although the largest number of swabs taken was in the North West of England (*n* = 24).

Asymptomatic swabbing uptake was higher for exposures where antiviral use was reported (52.0%) compared to those without reported antiviral use (12.5%).

There was one detection of Influenza A(H5) related to an individual exposed at an IP, although it should be noted that this individual had very prolonged and unusually high levels of exposure.^10^

Univariable analysis demonstrated reduced likelihood of asymptomatic swabbing with exposures related to WB detections of AI (OR 0.46, 95% CI:0.27–0.78) and a trend to reduced likelihood for being part of the category of workers not in disease control activities (OR 0.67, 95% CI:0.39–1.14).

This analysis also identified an increased likelihood for asymptomatic swabbing in the South West region of England (OR 5.07, 95% CI:1.66–17.0) and for those who received prophylaxis (OR 7.58, 95% CI:4.21–14.2).

## 4 DISCUSSION

This pilot has demonstrated that asymptomatic testing for surveillance and case detection is feasible and, despite small participant numbers, detected avian influenza A(H5N1) in a person from the South West of England. This was described elsewhere,^10^ but the detection occurred in an individual with confirmed close contact with infected avian species without PPE; health surveillance did not detect A(H5N1) in any other individuals associated with the same incident or with direct exposure to the case. This was the first human detection of A(H5) in England and would not have been detected in the absence of this pilot study.

Regional association with likelihood of asymptomatic swabbing may be explained by specific local factors and barriers. Increased uptake in the South West may be due to local awareness following the reported human case in the region. Additionally, the South West were proactive adopters of the pilot and used a postal system early on which was found to be successful. The higher uptake in the South West demonstrates that, if adopted as a surveillance strategy, there are opportunities to increase overall uptake of asymptomatic testing.

A reduced likelihood in uptake in those exposed to WB detections was expected. The extent of these exposures often varies, and individuals involved are from a wider range of backgrounds (including non‐occupational exposures) who are likely to have perceived the risk of exposure differently. Notification of EPs exposed at WB incidents was often delayed beyond the 10‐day follow‐up period as WB testing is carried out for surveillance purposes, unlike the rapid testing of IPs, reducing the eligible cohort in this group.

The proportions of those eligible for this pilot who accepted the swabbing were relatively low for both IP and WB detections. However, the association with EPs who received antiviral prophylaxis, may indicate that those who use antivirals are potentially more accepting of public health interventions.

A limitation to this pilot was that workload pressures restricted the number of surveillance forms that could be returned by HPTs impacting amount of information available for this analysis, in terms of both data quality and reporting of swabs taken. However, as the same constraints affect organisation of asymptomatic swabbing, it is unlikely to impact the identification of asymptomatic swabbing.

Due to the eligibility criteria, it should be noted that it is not representative of EPs in general. For example, PPE use should have been higher in trained animal health disease control staff and therefore less likely to be eligible for inclusion; although not explored here, additional studies are assessing asymptomatic infection among PPE users.

Self‐swabbing was the most practical sampling technique for logistical reasons in most of these scenarios; while this may have less assurance than sample collection by trained health care workers, several epidemiological surveys have successfully employed this approach for other respiratory virus infections.

Qualitative research is being undertaken to assess the underlying factors such as knowledge and attitudes as well as logistical and workload challenges associated with the low uptake in different regions and incidents in order to improve future uptake of asymptomatic self‐swabbing.

## 5 CONCLUSION

Following a human detection of A(H5N1) through this pilot, surveillance has detected other infections among EPs in the United States and Spain. In the context of high burden of disease in birds and the need to better understand risk to humans, we recommend the introduction of an asymptomatic testing programme, which has also since been included in updated recommendations from WHO.^1^ Further work is needed to develop the system including incorporating behavioural evidence to increase uptake. This will help identify early emergence of AI viruses from birds to humans and inform public health responses at an earlier stage.

## AUTHOR CONTRIBUTIONS

Fernando Capelastegui, Gavin Dabrera, Isabel Oliver, Jharna Kumbang and Clare Humphreys contributed to the study conception and design. Data collation, preparation and analysis were performed by Fernando Capelastegui and Julianna Smith. Laboratory data were prepared and processed by Andrew Mumford and Nick Richardson. The first draft of the manuscript was written by Fernando Capelastegui and Gavin Dabrera, and all authors commented on successive versions of the manuscript. All authors read and approved the final manuscript.

## CONFLICT OF INTEREST STATEMENT

No authors declare any conflicts of interest.

### PEER REVIEW

The peer review history for this article is available at https://www.webofscience.com/api/gateway/wos/peer-review/10.1111/irv.13187.

## Data Availability

No data are available. This work is carried out under Regulation 3 of The Health Service (Control of Patient Information) (Secretary of State for Health, 2002) (3) using patient identification information without individual patient consent. Data cannot be made publicly available for ethical and legal reasons, that is, public availability would compromise patient confidentiality as data tables list single counts of individuals rather than aggregated data.
